# Health and Economic Outcomes Associated With COVID-19 in Women at High Risk of HIV Infection in Rural Kenya

**DOI:** 10.1001/jamanetworkopen.2021.13787

**Published:** 2021-06-17

**Authors:** Nolan M. Kavanagh, Noora Marcus, Risper Bosire, Brian Otieno, Elizabeth F. Bair, Kawango Agot, Harsha Thirumurthy

**Affiliations:** 1 Perelman School of Medicine, Department of Medical Ethics and Policy, University of Pennsylvania, Philadelphia; 2 Center for Health Incentives and Behavioral Economics, University of Pennsylvania, Philadelphia; 3 Impact Research and Development Organization, Kisumu, Kenya

## Abstract

**Importance:**

COVID-19 lockdowns may affect economic and health outcomes, but evidence from low- and middle-income countries remains limited.

**Objective:**

To assess the economic security, food security, health, and sexual behavior of women at high risk of HIV infection in rural Kenya during the COVID-19 pandemic.

**Design, Setting, and Participants:**

This survey study of women enrolled in a randomized trial in a rural county in Kenya combined results from phone interviews, conducted while social distancing measures were in effect between May 13 and June 29, 2020, with longitudinal, in-person surveys administered between September 1, 2019, and March 25, 2020. Enrolled participants were HIV-negative and had 2 or more sexual partners within the past month. Surveys collected information on economic conditions, food security, health status, and sexual behavior. Subgroup analyses compared outcomes by reliance on transactional sex for income and by educational attainment. Data were analyzed between May 2020 and April 2021.

**Main Outcomes and Measures:**

Self-reported income, employment hours, numbers of sexual partners and transactional sex partners, food security, and COVID-19 prevention behaviors.

**Results:**

A total of 1725 women participated, with a mean (SD) age of 29.3 (6.8) years and 1170 (68.0%) reporting sex work as an income source before the COVID-19 pandemic. During the pandemic, participants reported experiencing a 52% decline in mean (SD) weekly income, from $11.25 (13.46) to $5.38 (12.51) (difference, −$5.86; 95% CI, −$6.91 to −$4.82; *P* < .001). In all, 1385 participants (80.3%) reported difficulty obtaining food in the past month, and 1500 (87.0%) worried about having enough to eat at least once. Reported numbers of sexual partners declined from a mean (SD) total of 1.8 (1.2) partners before COVID-19 to 1.1 (1.0) during (difference, −0.75 partners; 95% CI, −0.84 to −0.67 partners; *P* < .001), and transactional sex partners declined from 1.0 (1.1) to 0.5 (0.8) (difference, −0.57 partners; 95% CI, −0.64 to −0.50 partners; *P* < .001). In subgroup analyses, women reliant on transactional sex for income were 18.3% (95% CI, 11.4% to 25.2%) more likely to report being sometimes or often worried that their household would have enough food than women not reliant on transactional sex (*P* < .001), and their reported decline in employment was 4.6 hours (95% CI, −7.9 to −1.2 hours) greater than women not reliant on transactional sex (*P* = .008).

**Conclusions and Relevance:**

In this survey study, COVID-19 was associated with large reductions in economic security among women at high risk of HIV infection in Kenya. However, shifts in sexual behavior may have temporarily decreased their risk of HIV infection.

## Key Points

**Question:**

How have COVID-19 lockdowns factored into the economic well-being, food security, and sexual behavior of vulnerable populations in low- and middle-income countries?

**Findings:**

In this survey study of 1725 women at high risk of HIV infection in rural Kenya, COVID-19 lockdowns were associated with declines in employment, income, and numbers of sexual partners and transactional sex partners. Respondents also reported high levels of food insecurity.

**Meaning:**

These findings suggest that COVID-19 lockdowns may negatively impact the economic well-being of vulnerable populations with limited access to social services but may also temporarily reduce the risk of HIV transmission in these high-risk populations.

## Teaser

This survey study of women at high risk of HIV infection in a rural county of Kenya examines the association of COVID-19 lockdowns with declines in employment, income, and levels of food insecurity.

## Introduction

The COVID-19 pandemic poses a significant threat to various indicators of human well-being in low-income countries.^1^ For many countries in Africa, in particular, there is a significant risk that the pandemic and its economic consequences will erase years of progress in reducing poverty and malnutrition.^2^ Similarly, it may also challenge efforts to combat highly prevalent infectious diseases, such as HIV/AIDS.^3,4^ However, despite broad concern, relatively few published studies have quantified the size and nature of the pandemic’s impact in Africa.

Early in the pandemic, countries in Africa had substantially fewer COVID-19 cases and deaths per capita than countries in other parts of the world.^2^ However, waves of infection have since brought the continent to over 2.7 million cases and 65 000 deaths at the end of 2020.^5^ The pandemic has already harmed economic well-being and health outcomes.^1^ Individual preventive behaviors and strict social distancing measures introduced by governments may have slowed the spread of COVID-19 but reduced economic activity. Low- and middle-income countries (LMICs) have also been affected by the closure of international borders and the large contraction in the global economy, further reducing economic activity.

Given these multifaceted disruptions to well-being, there is a vital need to understand how the pandemic has affected individuals in LMICs, especially the most vulnerable populations in these countries. Researchers have called for greater attention to adolescents and young women in Africa,^6^ who are vulnerable to social and educational disruptions. For example, reports of online human trafficking in Kenya spiked amidst lockdowns in mid-2020.^7^ Sex workers are especially vulnerable to COVID-19, given their lack of social and institutional support.^8^ Data on how the pandemic has affected social and economic conditions for young women can provide insight into immediate and long-term consequences while informing appropriate policy responses.

In this study, we aimed to evaluate the health and economic consequences associated with COVID-19 among a population of women at high risk of HIV infection. Using cross-sectional and longitudinal data from rural Kenya, we examined economic outcomes, such as income and food insecurity, to assess the experiences of vulnerable populations during COVID-19, as well as health outcomes, such as sexual risk behavior, to provide an important early indication of how the pandemic may interact with the HIV epidemic in high-prevalence countries throughout Africa.

## Methods

### Study Setting and Participants

Data were collected from participants in a cluster randomized trial (ClinicalTrials.gov identifier NCT03135067) of an HIV self-testing intervention among women at high risk of HIV infection in Kenya. The parent study, described elsewhere,^9^ enrolled 2090 women from 66 geographic clusters in the rural and peri-urban communities of Siaya County, Kenya, a region with the country’s third-highest prevalence of HIV (15.3%) and high HIV/STI incidence.^9^ Women were eligible if they were aged 18 years or older, HIV negative, and had reported 2 or more male sexual partners in the past 4 weeks, thus representing a population at high risk of HIV infection. The present study and the parent study were approved by institutional review boards at the University of Pennsylvania and Maseno University. Participants provided written informed consent. This article followed the American Association for Public Opinion Research (AAPOR) reporting guideline.

### Data Collection

Between June 2017 and August 2018, 2090 participants were enrolled in the parent study. At baseline, they were administered a questionnaire about their demographic and socioeconomic characteristics, sexual behavior, and HIV testing history. Baseline data on education, marital status, and transactional sex as a primary or secondary source of income were used in the present study. Every 6 months, participants were administered follow-up questionnaires on their health and economic well-being until March 25, 2020, when the parent study ended. Participants’ most recent follow-up data on monthly income and numbers of sexual partners and transactional sex partners were used in the present study if collected on or after September 1, 2019, thereby constituting a 7-month pre–COVID-19 period.

Between May 13 and June 29, 2020, 1725 participants were successfully contacted by phone and administered a posttrial COVID-19 survey, for a response rate of 82.5%. This survey assessed income, employment hours (during the week before the survey and a recall of a typical week before lockdowns), food security, concern about COVID-19 symptoms, numbers of sexual partners and transactional sex partners, and engagement in COVID-19 prevention measures. Respondents were not offered incentives. The survey is provided as the eAppendix in the Supplement. Its distribution coincided with a period when various transmission prevention measures were put in place, thereby constituting the COVID-19 period of the study. On March 15, 2020, the Kenyan government implemented a series of COVID-19 policy responses by closing schools and international borders and by imposing strict physical distancing measures, such as a nightly curfew and limits on the size of public gatherings.^10^ The government also halted domestic flights, limited movement into and out of emerging COVID-19 hotspots, and urged people to work from home.

### Outcomes

We analyzed several outcomes, some assessed only in the COVID-19 survey, and others assessed longitudinally using data collected before the pandemic. Longitudinal analyses comprised changes in self-reported income and numbers of sexual partners and transactional sex partners. As for income, participants reported monthly income in the pre–COVID-19 surveys and weekly income during COVID-19; the 2 were harmonized by dividing prepandemic income by 4. All incomes were then converted to USD using the average exchange rate from May 13 to June 29, 2020 (0.0094 KES per US $1). Transactional sex was defined as any sex in exchange for money, goods, or services during the past month. The sexual behavior outcomes served as important indicators of the substantial HIV risk faced by participants.^11^ The outcomes assessed only in the COVID-19 survey included food security, expectations about economic status in the next 6 months, general health, health concerns that participants had for themselves and for their loved ones, experience of COVID-19 symptoms, and access to health care. Using the COVID-19 survey, we also assessed changes in weekly employment hours, collected as self-reported hours worked in the week before the survey and a recall of a typical week before the COVID-19 lockdowns.

### Statistical Analysis

Descriptive analyses were used to examine outcomes assessed only in the COVID-19 survey. For longitudinal analyses, we defined the pre–COVID-19 period using data from participants’ most recent follow-up survey in the parent study, conducted between September 1, 2019, and March 25, 2020. Participants were excluded from longitudinal analyses (but not cross-sectional ones during COVID-19) if they did not complete a follow-up survey during this window. For these comparisons, we calculated changes in income and sexual behavior and applied ordinary least squares regressions with standard errors clustered by study cluster. The change in participants’ employment hours was similarly analyzed, except that these data were collected only in the COVID-19 survey by recall. The criterion for statistical significance was set at *P* < .05 in 2-sided tests. Subgroup analyses compared trends in health and economic outcomes for participants with different levels of educational attainment (primary or less vs secondary or more) and baseline engagement in sex work (as a primary or secondary source of income vs not) using ordinary least squares models clustered by study cluster. All statistical analyses were performed in R version 3.6.3 (R Project for Statistical Computing); clustered regressions used the R package “miceadds” version 3.10.

## Results

Between June 2017 and August 2018, 2090 participants were enrolled in the parent study. Follow-up continued until March 25, 2020, with a mean follow-up duration of 19.1 months. Between May 13 and June 29, 2020, 1725 participants (82.5%) were administered the COVID-19 phone survey. Among them, 1504 had also been administered a follow-up survey in the 7 months before COVID-19 and, therefore, had longitudinal data. At baseline, 1258 (73.1%) participants who completed a COVID-19 survey were married or cohabitating (Table 1). Nearly all participants in the parent study (approximately 95%) reported ever engaging in transactional sex^9^; 266 (15.5%) reported that sex work was their primary source of income, and an additional 904 (52.5%) reported that sex work was a secondary source. At the time of the COVID-19 survey, participants’ mean (SD) age was 29.3 (6.8) years.

**Table 1.** Baseline Demographics of Participants and Prevention Behaviors During COVID-19

Characteristics	All respondents, No. (%) (N = 1725)^a^
Age, mean (SD), y^b^	29.3 (6.8)
Education^c^	
Some primary or less	535 (31.1)
Primary	516 (30.0)
Some secondary	330 (19.2)
Secondary	276 (16.0)
Postsecondary	64 (3.7)
Marital status^c^	
Married or cohabitating	1258 (73.1)
Single	324 (18.8)
Separated, divorced, or widowed	139 (8.1)
Sex work^c^	
As primary source of income	266 (15.5)
As secondary source of income	904 (52.5)
Attended meeting or gathering with >5 persons in the last wk^b^	182 (10.6)
Went to a restaurant, bar, club, etc, in the last wk^b^	50 (2.9)
Went to a religious gathering in the last wk^b^	10 (0.6)
Attended a wedding in the last 14 d^b^	7 (0.4)
Attended a funeral in the last 14 d^b^	147 (8.5)
Rode a public vehicle or bus with >3 persons in the last wk^b^	318 (18.4)
Went to a beauty parlor, etc, in the last wk^b^	335 (19.4)
Went to a market in the last wk^b^	1354 (78.5)

^a^ Percentages taken of nonmissing responses.

^b^ During the COVID-19 pandemic.

^c^ At baseline enrollment.

Participants reported high levels of engagement in COVID-19 prevention behaviors. During the week prior to the phone survey, only 182 (10.6%) had attended a gathering with 5 or more people, 50 (2.9%) had attended a social event (such as going to a restaurant, bar, club, party, or concert), and 10 (0.6%) had attended a religious gathering (Table 1). During the 14 days prior to the survey, 147 (8.5%) of respondents had attended a funeral and 7 (0.4%) had attended a wedding. Engagement in other behaviors was higher, with 318 participants (18.4%) having been in a public vehicle or bus with more than 3 people, 335 (19.4%) having been to a beauty parlor (or similar establishment), and 1354 (78.5%) having gone to a market, all assessed in the past week. However, 1354 (84.3%) reported having no physical contact, such as shaking hands or hugging, with anyone outside their household during the previous week (eTable in the Supplement).

**Figure 1.**
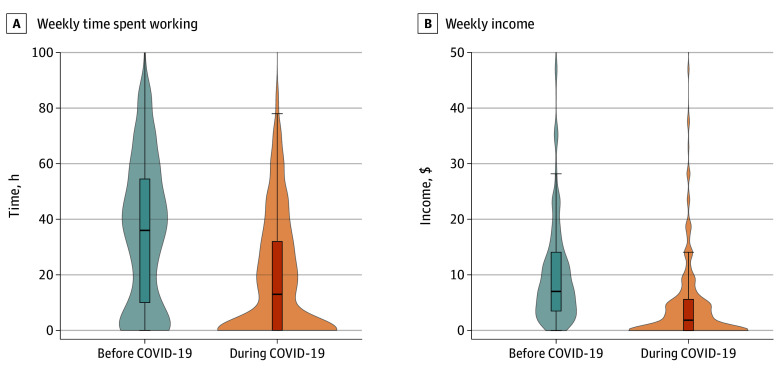
Weekly Hours Worked and Income Earned Before and During COVID-19 Employment hours were assessed only in the COVID-19 survey as hours worked in the previous week plus a recall of a typical week before lockdowns (1671 participants). Income was assessed longitudinally in surveys both before and during COVID-19 (1502 participants). Boxes represent the interquartile range (IQR), lines within the boxes represent the median, and whiskers extend 1.5 × IQR from the upper or lower hinge, or to the highest or lowest value if no values are beyond 1.5 × IQR. Shaded areas represent the distributions of values. Hours above 100 and income above $50 are not shown but are included in statistics derived from these variables.

Economic outcomes associated with the pandemic were assessed in the COVID-19 survey and longitudinally. Participants’ incomes declined significantly once the pandemic began, from a mean (SD) of US $11.25 (13.46) per week before COVID-19 to US $5.38 (12.51) per week during COVID-19, a 52% decline (difference, −$5.86; 95% CI, −$6.91 to −$4.82) (Figure 1). These declines were not evenly distributed among participants, as the median weekly income fell from $7.05 to $1.88. In the COVID-19 survey, 1093 (66.9%) participants reported that their earnings were lower than usual (eTable in the Supplement). Consistent with the decline in income, participants also reported significantly less employment. They recalled working a mean (SD) 35.5 (26.3) hours per week before lockdowns, but in the week prior to the COVID-19 survey they worked 19.1 (21.8) hours, for a 46% decline in employment hours (difference, −16.4 hours; 95% CI, −17.9 to −14.9 hours) (Figure 1). Participants also had a negative economic outlook for the future, with 1062 (68.3%) expecting they would be “somewhat worse off” or “much worse off” 6 months later (eTable in the Supplement).

Consistent with the decline in economic well-being, participants also reported high levels of food insecurity. A total of 1385 participants (80.3%) reported difficulty obtaining food during the previous month, and 1500 (87.0%) worried at least once during the past month that their household would not have enough food to eat (Table 2). Over one-quarter of participants worried about not having enough food more than 10 times in the past month (450 participants [26.1%]). Comparably large proportions of respondents reported being frequently unable to eat the kinds of foods they preferred because of a lack of resources (eg, 383 participants [22.2%] unable to eat preferred foods >10 times). During the previous month, nearly half (825 [47.8%]) of participants reported that they or another household member had gone to sleep hungry at least once for lack of food. Participants also expressed high levels of concern about their health and that of their loved ones. During the previous week, 794 (46.0%) were moderately or extremely worried about their own current or future health, and 977 (56.6%) were comparably worried about the health of their loved ones (Table 2).

**Table 2.** Food Insecurity and Health Measures, Assessed During COVID-19

Characteristics	All respondents, No. (%) (n = 1725)^a^
**Difficulty obtaining food in last month**	
Yes	1385 (80.3)
No	339 (19.7)
**Worried that household would not have enough food in last month**	
Never	225 (13.0)
Rarely (1-2 times)	475 (27.5)
Sometimes (3-10 times)	575 (33.3)
Often (>10 times)	450 (26.1)
**Self or household member unable to eat preferred foods in last month**	
Never	233 (13.5)
Rarely (1-2 times)	495 (28.7)
Sometimes (3-10 times)	612 (35.5)
Often (>10 times)	383 (22.2)
**Self or household member went to sleep hungry in last month**	
Never	900 (52.2)
Rarely (1-2 times)	513 (29.7)
Sometimes (3-10 times)	270 (15.7)
Often (>10 times)	42 (2.4)
**Worry about own current or future health in the last week**	
Not at all worried	306 (17.7)
Slightly worried	625 (36.2)
Moderately worried	508 (29.4)
Extremely worried	286 (16.6)
**Worry about health of loved ones in the last week**	
Not at all worried	313 (18.1)
Slightly worried	435 (25.2)
Moderately worried	501 (29.0)
Extremely worried	476 (27.6)

^a^ Percentages taken of nonmissing responses.

**Figure 2.**
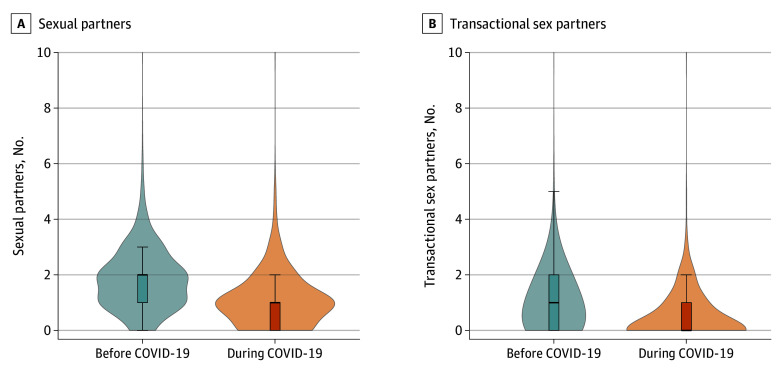
Sexual Behavior in the Past Month, Before and During COVID-19 Numbers of sexual partners (1504 participants) and transactional sex partners (1500 participants) were assessed longitudinally in surveys both before and during COVID-19. Boxes represent the interquartile range (IQR), lines within the boxes represent the median, and whiskers extend 1.5 × IQR from the upper or lower hinge, or to the highest or lowest value if no values are beyond 1.5 × IQR. Shaded areas represent the distributions of values. Values above 10 are not shown in either graph but are included in statistics derived from these variables.

Sexual risk behavior declined considerably during the COVID-19 lockdowns. In surveys before the pandemic, 1383 (92.0%) of participants with longitudinal data had reported having sex with at least 1 person in the previous month, for a mean (SD) of 1.8 (1.2) partners, including participants who were not sexually active (Figure 2). During the pandemic, however, the rate dropped to 1081 (71.9%) and the mean number of partners declined to 1.1 (1.0) (−0.75 sexual partners; 95% CI, −0.84 to −0.67). With respect to transactional sex, the declines were even greater. Before the pandemic, 912 (60.8%) of the participants had reported engaging in any transactional sex in the past month, with a mean (SD) 1.0 (1.1) transactional sex partner per participant. During the pandemic, the rate halved to 465 (31.0%) engaging in any transactional sex and a mean (SD) of 0.5 (0.8) transactional sex partners (−0.57 transactional sex partners; 95% CI, −0.64 to −0.50).

**Figure 3.**
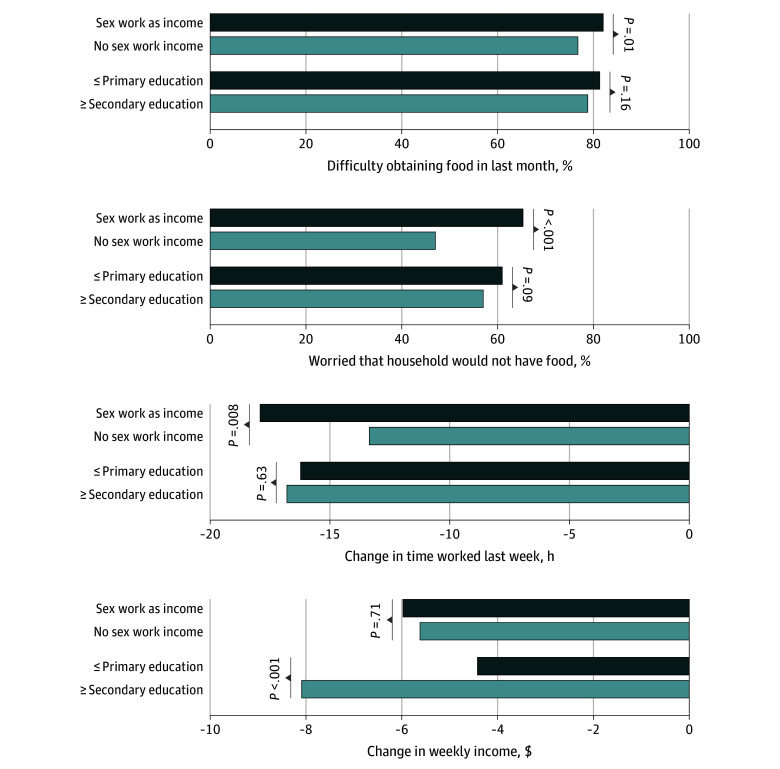
Food Security and Economic Outcomes by Education and Reliance on Sex Work for Income Prior to COVID-19 Each *P* value indicates the results from an unadjusted ordinary least squares regression with robust standard errors clustered by study cluster, comparing the indicated outcome between the 2 subgroups (using participants’ baseline data). The second panel assesses the percentage of participants that were sometimes or often worried that their household would not have enough food in the last month. Sample sizes are as follows: difficulty obtaining food (1720 participants responding), worrying about having enough food (1721 participants), change in weekly employment hours (1667 participants), and change in weekly income (1498 participants).

In subgroup analyses, participants who relied on transactional sex for income were 5.3% more likely to report difficulty obtaining food in the last month, compared with those not reliant on transactional sex (959 [82.0%] vs 423 [76.8%]; 95% CI, 1.2% to 9.3%). Participants reliant on transactional sex were also 18.3% more likely to report being sometimes or often worried that their household would have enough food (764 [65.3%] vs 259 [47.0%]; 95% CI, 11.4% to 25.2%) (Figure 3). Similarly, they reported a significantly larger decline in weekly employment (−17.9 vs −13.4 hours, or 4.6 fewer hours per week; 95% CI, −7.9 to −1.2). However, they did not report a significantly greater decline in weekly income (−$5.98 vs −$5.62, or a difference of −$0.35; 95% CI, −$2.21 to $1.50).

Participants reporting difficulty obtaining food in the last month were not significantly different by level of education (854 [81.3%] for primary or less education vs 528 [78.8%] for secondary or more, a difference of 2.5%; 95% CI, −1.0% to 6.1%) (Figure 3). With respect to economic status, the 2 groups of women experienced essentially identical changes in weekly working hours (−16.2 hours vs −16.8 hours, or 0.6 more hours; 95% CI, −1.7 to 2.9). However, while both groups experienced declines in weekly income, women with less education reported a smaller decline than their better-educated counterparts by $3.66 (−$4.44 vs −$8.09; 95% CI, $2.20 to $5.11).

## Discussion

The COVID-19 pandemic was associated with worse economic outcomes among a population of low-income women who are reliant on transactional sex for income and who face a high risk of HIV infection. During this period, the women also experienced high levels of food insecurity and psychological burden in the form of concern for their health and that of their loved ones. However, the pandemic was associated with decreased engagement in sexual risk behaviors as well, which could be driven by social distancing measures or reduced demand for transactional sex.

The COVID-19 pandemic has had substantial economic consequences in Kenya, especially for the high-risk population that we studied. The Kenyan economy contracted by an estimated 5.6% amidst lockdowns, curfews, and other COVID-19 prevention measures from April through June 2020.^12,13^ By comparison, the women who participated in our study faced a nearly 10-fold greater decline in income, an indication of the pandemic’s disproportionate economic impact on low-income, vulnerable populations. Moreover, over two-thirds of participants expected their economic condition to continue to decline over the next 6 months. The evidence from this study underscores the importance of considering and mitigating inequitable outcomes of COVID-19 and its policy responses, such as lockdowns and strict physical distancing measures.^14,15^

While there is limited evidence from nationally representative surveys on the economic impacts of COVID-19 in LMICs, our results are consistent with several existing studies. Surveys of heterogeneous populations across 9 LMICs, 5 of which were in Africa, documented declines in income up to 87% and rises in joblessness up to 49%, especially among populations of lower socioeconomic status.^16^ In a survey of community health workers, small business owners, and vulnerable community members in rural Kenya, nearly all respondents reported a decline in income.^17^ Similarly, over two-thirds of cell phone users across rural and urban Kenya and Uganda reported decreases in income-generating activities.^18^ Other surveys in vulnerable populations, such as low-income mothers in Kenya^19^ and mothers in rural Bangladesh,^20^ have documented double-digit percentage declines in income. Also, low-income and vulnerable populations in high-income countries have experienced inequitable cuts to economic opportunity.^21^ Indeed, early in the pandemic, many experts expected that marginalized communities would be disproportionately affected, even in LMICs.^14,22^

In our survey of high-risk women, high rates of food insecurity coincided with substantial declines in income and employment. A high percentage of respondents worried about having enough to eat, went to sleep hungry, had difficulty obtaining food, and negatively adjusted their consumption patterns. While some surveys in Africa have documented economic disruptions caused by COVID-19 without changes in food security,^19,23^ the bulk of surveys in LMICs suggest that the two tend to move in parallel. For example, over four-fifths of respondents in rural Meru County, Kenya, reported an inability to afford sufficient food because of reduced household income.^17^ Surveys in Kenya and Uganda have documented nearly 50 percentage-point longitudinal increases in respondents worrying about having enough food, with lower-income households especially likely to suffer poorer consumption.^18^ Among households in rural Bangladesh, rates of food insecurity have increased 6-fold during the pandemic.^20^ Our results are in line with these reports.

Our findings highlight how COVID-19 has been associated with reductions in economic security for marginalized populations within LMICs, especially sex workers. Sex workers are at greater risk of the consequences of COVID-19 because they experience lower social and economic support, stigma, higher risk of infection inherent in their work, and more.^8^ However, the needs of sex workers during the pandemic have been neither well studied nor well attended to by policy makers. The population of vulnerable women that we surveyed experienced considerable food insecurity and declines in economic security. These burdens were felt even more greatly by the women who relied on transactional sex as a primary or secondary source of income. A lesser degree of insecurity was experienced by women with less formal education, underscoring the particular social and economic vulnerability of women who are reliant on transactional sex as an income source.

However, the health consequences of COVID-19 for women reliant on transactional sex may be blunted by shifts in sexual behavior. Participants reported fewer sexual partners and transactional sex partners than before COVID-19. Several mechanisms may explain the large declines in both transactional sex and income. These include mobility restrictions, closures of bars and other venues where transactional sex is common, women’s engagement in COVID-19 preventive behaviors, or reduced demand from male partners who may have been engaging in COVID-19 preventive behaviors or experiencing income declines themselves. There have been reports of decreased demand for sex work in Kenya during the pandemic,^22^ and the women in our study reported a high degree of engagement in prevention behaviors—more than 8 in 10 reported no physical contact with anyone outside their household in the week before the survey. Because these women were enrolled in the parent trial for their high risk of HIV, these shifts in sexual behavior may have resulted in a short-term decline in risk of HIV transmission. Even so, the provision of HIV prevention services during the pandemic requires attention by policy makers. For example, other researchers have documented dramatic declines in HIV testing in other parts of Africa during the pandemic, and there is continued concern about the continuity of care during lockdowns and economic crises.^24^ Over time, food insecurity and loss of income among low-income women may contribute to greater engagement in transactional sex, as several studies in Africa have shown that poverty and economic shocks increase women’s engagement in sex work.^11,25^ These shocks may ultimately raise their risk of acquiring HIV.

### Limitations

Our study has several important limitations. Some outcomes, such as a food insecurity, were assessed at only 1 time point, so we cannot determine the consequences of the pandemic on them. Our longitudinal outcomes documented the consequences of COVID-19 but were evaluated early on in the pandemic. More data are necessary to track long-term outcomes associated with the pandemic. However, considerable anecdotal evidence from Africa suggests that the consequences reported in our study have been sustained over time. Second, the decline in transactional sex observed in our study may not persist. Prolonged economic hardship may, instead, lead to an increased reliance on transactional sex for income. Since our study was conducted, many countries (including Kenya) have introduced social protection measures such as cash transfer programs that may mitigate the impacts on economic and food insecurity. That said, sex workers routinely experience diminished access to social resources,^8^ so the effects of the pandemic on their health and socioeconomic outcomes warrant continued study. Third, the COVID-19 telephone survey may have been subject to selection bias and recall bias, especially for outcomes assessed by more remote recall.

## Conclusions

This study informs recent and ongoing debates over policy measures to address COVID-19 in LMICs. The association of COVID-19 with employment and income outcomes and the high rates of food insecurity among low-income women in Kenya illustrate the risks to economic well-being posed by both the pandemic and its policy responses. Particularly in LMICs with high underlying poverty and food insecurity, policy makers must consider and mitigate disruptions to economic security, especially for vulnerable populations. Additionally, the declines in sexual behavior may shed light on one way that COVID-19 could influence the HIV/AIDS epidemic in sub-Saharan Africa. Continued study is necessary to determine whether these changes in sexual behavior persist.

## Notes

Supplement. **eFigure.** Study Timeline and Flow of Participants **eTable.** Additional Economic and Health Outcomes During COVID-19 **eAppendix.** COVID-19 Phone Survey With Jikinge Participants
