# Supplementary material for: Health and Economic Outcomes Associated With COVID-19 in Women at High Risk of HIV Infection in Rural Kenya
Source: JAMA Netw Open. 2021 Jun 17;4(6):e2113787. doi: 10.1001/jamanetworkopen.2021.13787 (PMC12578490; doi:10.1001/jamanetworkopen.2021.13787)
Supplement: Supplement. — eFigure. Study Timeline and Flow of Participants eTable. Additional Economic and Health Outcomes During COVID-19 eAppendix. COVID-19 Phone Survey With Jikinge Participants [file jamanetwopen-e2113787-s001.pdf]

Supplemental Online Content

Kavanagh NM, Marcus N, Bosire R, et al. Health and economic outcomes associated with COVID-19 in women at high risk of HIV infection in rural Kenya. *JAMA Netw Open.* 2021;4(6):e2113787. doi:10.1001/jamanetworkopen.2021.13787

eFigure. Study Timeline and Flow of Participants

eTable. Additional Economic and Health Outcomes During COVID-19

eAppendix. COVID-19 Phone Survey With Jikinge Participants

This supplemental material has been provided by the authors to give readers additional information about their work.

© 2021 Kavanagh NM et al. *JAMA Network Open.*

eFigure. Study Timeline and Flow of Participants

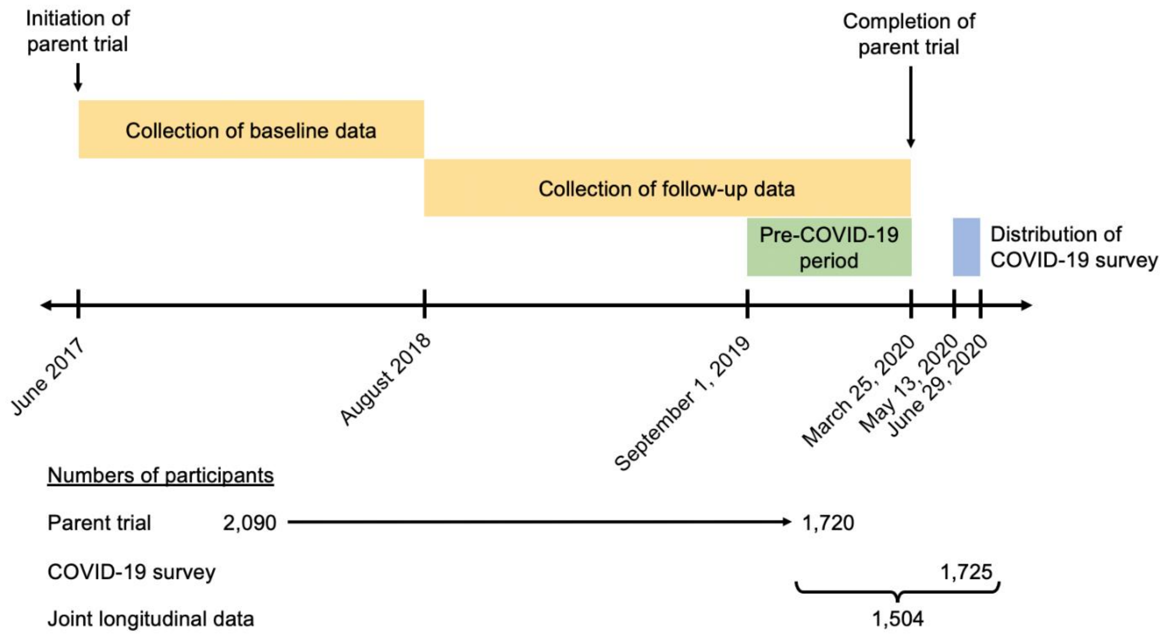

© 2021 Kavanagh NM et al. *JAMA Network Open.*

eTable. Additional Economic and Health Outcomes During COVID-19

	**All respondents** n=1,725
**Overall health rating**	
Very good	58 (3)
Good	518 (30)
Fair	928 (54)
Poor	220 (13)
**Participant had COVID-19 symptoms in last week**	
Yes	427 (25)
No	1,298 (75)
**Household member(s) had COVID-19 symptoms in last week**	
Yes	281 (18)
No	1,313 (82)
**Missed clinic or key populations drop-in center appointment in last month**	
Yes	65 (5)
No	1,403 (95)
**Last physical contact**	
Today	88 (5)
Last 2 days	103 (6)
Last 3 to 6 days	62 (4)
More than 1 week ago	367 (23)
None outside household	987 (61)
**Expected household economic status in 6 months**	
Much better off than now	57 (4)
Somewhat better off than now	342 (22)
Same	94 (6)
Somewhat worse off than now	439 (28)
Much worse off than now	623 (40)
**Earnings now vs. typical week**	
Lower than usual	1,093 (67)
Similar	235 (14)
Higher than usual	306 (19)

Notes: Percentages taken of non-missing responses.

© 2021 Kavanagh NM et al. *JAMA Network Open.*

eAppendix. COVID-19 Phone Survey With Jikinge Participants

COVID-19 Phone Survey with Jikinge participants

No.	Question	Responses
1	Interviewer ID	___ ___
2	Date of Interview	___ ___/___ ___ ___/ ___ ___ ___ ___<br>D D M M M Y Y Y Y
3	Time of Interview (HH:MM)	__ __ : __ __
3	Participant ID	
4	Participant Name	
5	Cluster ID	__ __ __
6	Enter Name of Cluster	
7	Enter Name of Hotspot or Beach	

Introduction:
The coronavirus (also called Covid-19) is a virus that infects individuals around the world and can cause respiratory problems and in some cases, death. The Covid-19 is already circulating in Kenya, and many efforts are being invested to prevent its spread in our community. In that light, the Kenyan government took a number of measures to prevent the spread of Covid-19. People were asked to stay home, to avoid gatherings and traveling, and to frequently wash their hands. We would like to ask you a few questions about your experience during this time.

INTERVIEWER: RECORD ANSWER IN THE LAST COLUMN

© 2021 Kavanagh NM et al. *JAMA Network Open.*

**Section A. Demographic and socio-economic information**<br>INTERVIEWER: RECORD ANSWER IN THE LAST COLUMN			
**NO.**	**QUESTIONS**	**CODING CATEGORIES**	**RESPONSE**
1	I'd like to begin by asking, what is your age?	NUMBER OF YEARS (2 DIGITS)<br><br>98 DON'T KNOW	
2	Who do you currently live with?<br><br>READ ALL OPTIONS<br>A. My parents<br>B. My spouse/partner<br>C. My child(ren)<br>D. My sibling(s) or other relative(s)<br>E. Friends<br>F. I live alone	YES NO	
		PARENTS 1 2	
		SPOUSE 1 2	
		CHILDREN 1 2	
		RELATIVES 1 2	
		FRIENDS 1 2	
		ALONE 1 2	
3	Where do you live now?	1 WITHIN RARIEDA SUB COUNTY<br>2 WITHIN BONDO SUB COUNTY<br>3 WITHIN SIAYA SUB COUNTY<br>4 ELSEWHERE IN SIAYA COUNTY<br>5 KISUMU COUNTY<br>6 ANOTHER COUNTY IN KENYA (RURAL)<br>7 ANOTHER COUNTY IN KENYA (URBAN)<br>99 REFUSE TO ANSWER	
5	What are your housing conditions?	1 A HOUSE OR APARTMENT WITH GARDEN<br>2 A HOUSE OR APARTMENT WITHOUT GARDEN<br>3 AN APARTMENT WITH BALCONY<br>4 AN APARTMENT WITHOUT BALCONY<br>5 A ROOM<br>6 A HUT<br>7 A SHACK<br>8 HOMELESS<br><br>99 REFUSE TO ANSWER	

© 2021 Kavanagh NM et al. *JAMA Network Open.*

Section B. Daily life during the Coronavirus epidemic			
NO.	QUESTIONS	CODING CATEGORIES	RESPONSE
1	How many people apart from your household members did you talk to yesterday, face to face (not by phone, chat, etc.)	NUMBER OF PEOPLE (2 digits)<br><br>98 DON'T KNOW<br>99 REFUSE TO ANSWER	
2	When was the last time you shook hands, hugged someone, or had any form of physical contact with someone other than a household member?	1 TODAY<br>2 LAST TWO DAYS<br>3 LAST 3 TO 6 DAYS<br>4 MORE THAN ONE WEEK AGO<br>5 NO CONTACTS WITH PERSONS OUTSIDE MY HOUSEHOLD<br><br>98 DON'T KNOW<br>99 REFUSE TO ANSWER	
3	During the past month, did you have difficulties in obtaining food?	1 YES<br>2 NO<br>99 REFUSE TO ANSWER	
4	During the past month, did you worry that your household would not have enough food to eat?	1 NEVER<br>2 RARELY (1-2 TIMES)<br>3 SOMETIMES (3-10 TIMES)<br>4 OFTEN (10+ TIMES)<br>98 DON'T KNOW<br>99 REFUSE TO ANSWER	
5	During the past month, were you or any household member not able to eat the kinds of foods you preferred because of a lack of resources?	1 NEVER<br>2 RARELY (1-2 TIMES)<br>3 SOMETIMES (3-10 TIMES)<br>4 OFTEN (10+ TIMES)<br>98 DON'T KNOW<br>99 REFUSE TO ANSWER	
6	During the last month, did you or any household member go to sleep at night hungry because there was not enough food?	1 NEVER<br>2 RARELY (1-2 TIMES)<br>3 SOMETIMES (3-10 TIMES)<br>4 OFTEN (10+ TIMES)<br>98 DON'T KNOW<br>99 REFUSE TO ANSWER	
7	During the last month, have you suffered any form of violence of discrimination?<br><br>READ ALL OPTIONS<br><br>A. Physical violence at home<br><br>B. Physical violence outside home<br><br>C. Sexual violence<br><br>D. Discrimination because of my social/economic status<br><br>E. Discrimination because of my ethnicity or nationality<br><br>F. No violence or discrimination	SELECT ALL THAT APPLY<br><br>YES NO<br>VIOLENCEHOME 1 2<br><br>VIOLENCEOUT 1 2<br><br>VIOLENCESEXUAL 1 2<br><br>SOCIO-ECONOMIC 1 2<br><br>ETHNIC 1 2<br><br>NOVIOLENCE 1 2	

© 2021 Kavanagh NM et al. *JAMA Network Open.*

Section C. Economic outcomes and professional life during the Coronavirus epidemic			
NO.	QUESTIONS	CODING CATEGORIES	RESPONSE
1	What is your primary occupation?<br>*[Select respondent's self-reported category; do not read all choices]*	1 - Farmer who owns land<br>2 - Farmer who works on land owned by another<br>3 - Professional<br>4 - Clerical<br>5 – Sales and services<br>6 – Skilled manual<br>7 – Unskilled manual<br>8 – Domestic service<br>9 - Unemployed<br>10 - Student<br>11 - Other: ______________________<br>98: Don't know<br>99: Refuse to answer	
2	What were your working conditions like immediately before the COVID-related public health restrictions on work?	1 WORKER FROM HOME<br>2 WORKER IN AN OPEN SPACE (GARDENS, MARKET, SHOP, ROADSIDE, ETC)<br>3 WORKER IN A CLOSED INDOOR SPACE ALONE (OFFICE, ETC)<br>4 WORKER IN A CLOSED INDOOR SPACE WITH SEVERAL PEOPLE (OFFICE, ETC)<br>5 NOT APPLICABLE (JOBLESS, STUDENT)<br>98 DON'T KNOW<br>6 99 REFUSE TO ANSWER	
3	How many hours per week do you <u>usually</u> work (before COVID-19 restrictions)	NUMBER OF HOURS (3 digits)<br>98 DON'T KNOW<br>99 REFUSE TO ANSWER	
4	How many hours did you actually work in the past week?	NUMBER OF HOURS (3 digits)<br>98 DON'T KNOW<br>99 REFUSE TO ANSWER	
5	How much money did you earn in the past week? (Please do not count money earned by your family)	MONEY EARNED PER WEEK (IN SHILLINGS)<br>98 DON'T KNOW<br>99 REFUSE TO ANSWER	
6	Compared to a typical week (before the COVID-related shut-down), were your earnings in the past week…<br><br>[READ ALL 3 CHOICES]	1 HIGHER THAN USUAL<br>2 LOWER THAN USUAL<br>3 SIMILAR<br>98 DON'T KNOW<br>99 REFUSE TO ANSWER	
7	Compared to your household's current economic status, 6 months from now do you expect it to be…<br><br>[READ ALL 5 CHOICES]	1 MUCH BETTER OFF THAN NOW<br>2 SOMEWHAT BETTER OFF THAN NOW<br>3 SAME<br>4 SOMEWHAT WORSE OFF THAN NOW<br>5 MUCH WORSE OFF THAN NOW<br><br>98 DON'T KNOW<br>99 REFUSE TO ANSWER	
8	Are you working from home today?	1 YES<br>2 NO<br>3 NOT APPLICABLE (JOBLESS, NOT WORKING, STUDENT)<br>98 DON'T KNOW<br>99 REFUSE TO ANSWER	

© 2021 Kavanagh NM et al. *JAMA Network Open.*

Section D. Community prevention measures for coronavirus			
NO.	QUESTIONS	CODING CATEGORIES	RESPONSE
1	Were you in a meeting or gathering with more than 5 persons during the last seven days?	1 YES<br>2 NO<br><br>98 DON'T KNOW<br>99 REFUSE TO ANSWER	
2	Did you go to a restaurant, bar, club, dancing, party, or concert during the last seven days?	1 YES<br>2 NO<br><br>98 DON'T KNOW<br>99 REFUSE TO ANSWER	
3	Did you go to a religious gathering during the last seven days?	1 YES<br>2 NO<br><br>98 DON'T KNOW<br>99 REFUSE TO ANSWER	
4	Did you attend a wedding in the last 14 days?	1 YES, WITHIN THE LAST 7 DAYS<br>2 YES, WITHIN 8-14 DAYS<br>3 NO<br><br>98 DON'T KNOW<br>99 REFUSE TO ANSWER	
5	Did you attend a funeral in the last 14 days?	1 YES, WITHIN THE LAST 7 DAYS<br>2 YES, WITHIN 8-14 DAYS<br>3 NO<br><br>98 DON'T KNOW<br>99 REFUSE TO ANSWER	
6	Were you in a public vehicle or bus with more than 3 persons in the past seven days?	1 YES<br>2 NO<br><br>98 DON'T KNOW<br>99 REFUSE TO ANSWER	
7	Did you go to a beauty parlor, massage, spa, hairdresser, or nail studio in the past seven days?	1 YES<br>2 NO<br><br>98 DON'T KNOW<br>99 REFUSE TO ANSWER	
8	Did you go to a market in the past seven days?	1 YES<br>2 NO<br><br>98 DON'T KNOW<br>99 REFUSE TO ANSWER	
9	During the past 7 days, how worried or afraid were you about the health of your loved ones?<br>1=not worried to 5=extremely worried	1 NOT AT ALL WORRIED<br>2 SLIGHTLY WORRIED<br>3 MODERATELY WORRIED<br>4 EXTREMELY WORRIED<br>98 DON'T KNOW<br>99 REFUSE TO ANSWER	

© 2021 Kavanagh NM et al. *JAMA Network Open.*

Section E. Questions related to your personal health			
NO.	QUESTIONS	CODING CATEGORIES	RESPONSE
1	How would you rate your overall health?	1 Very Good<br>2 Good<br>3 Fair<br>4 Poor<br>98 Don’t Know<br>99 Refused to Answer	
2	Did you have any of the following health problems in the last 7 days: cough or sore throat, shortness of breath, headaches, body pains, fever, loss of taste or smell?	1 YES<br>2 NO<br><br>98 DON’T KNOW<br>99 REFUSE TO ANSWER	
3	Did any of your household members have any of the following health problems in the last 7 days: cough or sore throat, shortness of breath, headaches, body pains, fever, loss of taste or smell?	1 YES<br>2 NO<br><br>98 DON’T KNOW<br>99 REFUSE TO ANSWER	
4	During the past 7 days, how worried have you been about your current or future health?	1 NOT AT ALL WORRIED<br>2 SLIGHTLY WORRIED<br>3 MODERATELY WORRIED<br>5 EXTREMELY WORRIED	
5	Have you traveled to a clinic or health centre for any reason (to see a healthcare provider, collect medicines, go for a test, etc.) in the past 7 days?	1 YES<br>2 NO<br><br>99 REFUSE TO ANSWER	
6	Have you missed any clinic (or DICE) appointments in the past month?	1 YES<br>2 NO<br><br>98 DON’T KNOW<br>99 REFUSE TO ANSWER	
7	Have you had any difficulty in obtaining medicines in the past month, for either financial or non-financial reasons?	1 YES<br>2 NO<br><br>98 DON’T KNOW<br>99 REFUSE TO ANSWER	
8	Do you think you <u>will</u> have difficulty obtaining your medicines in the coming month, for either financial or non-financial reasons?	1 YES<br>2 NO<br><br>98 DON’T KNOW<br>99 REFUSE TO ANSWER	
9	During the past month, how many different sexual partners have you had?	NUMBER	
10	During the past month, how many men did you exchange sex for money, goods, food, housing or services?	NUMBER	
11	Overall, what has been the biggest challenge you have faced as a result of the coronavirus shut down measures in Kenya? (OPEN-ENDED RESPONSE)		
12	How many months do you think it will take you to economically recover from any impacts of the COVID-19 response in Kenya?		

**END TIME OF INTERVIEW (HH:MM)**	___ ___ : ___ ___

© 2021 Kavanagh NM et al. *JAMA Network Open.*
